# The Role of Apelin in Cardiovascular Diseases, Obesity and Cancer

**DOI:** 10.3389/fphys.2018.00557

**Published:** 2018-05-23

**Authors:** Marta B. Wysocka, Katarzyna Pietraszek-Gremplewicz, Dorota Nowak

**Affiliations:** Department of Cell Pathology, Faculty of Biotechnology, University of Wrocław, Wrocław, Poland

**Keywords:** apelin, apelin receptor, cardiovascular diseases, hypoxia, angiogenesis, obesity, diabetes, cancer

## Abstract

Apelin is an endogenous peptide identified as a ligand of the G protein-coupled receptor APJ. Apelin belongs to the family of adipokines, which are bioactive mediators released by adipose tissue. Extensive tissue distribution of apelin and its receptor suggests, that it could be involved in many physiological processes including regulation of blood pressure, body fluid homeostasis, endocrine stress response, cardiac contractility, angiogenesis, and energy metabolism. Additionally, this peptide participates in pathological processes, such as heart failure, obesity, diabetes, and cancer. In this article, we review current knowledge about the role of apelin in organ and tissue pathologies. We also summarize the mechanisms by which apelin and its receptor mediate the regulation of physiological and pathological processes. Moreover, we put forward an indication of apelin as a biomarker predicting cardiac diseases and various types of cancer. A better understanding of the function of apelin and its receptor in pathologies might lead to the development of new medical compounds.

## Introduction

**Figure 1:**
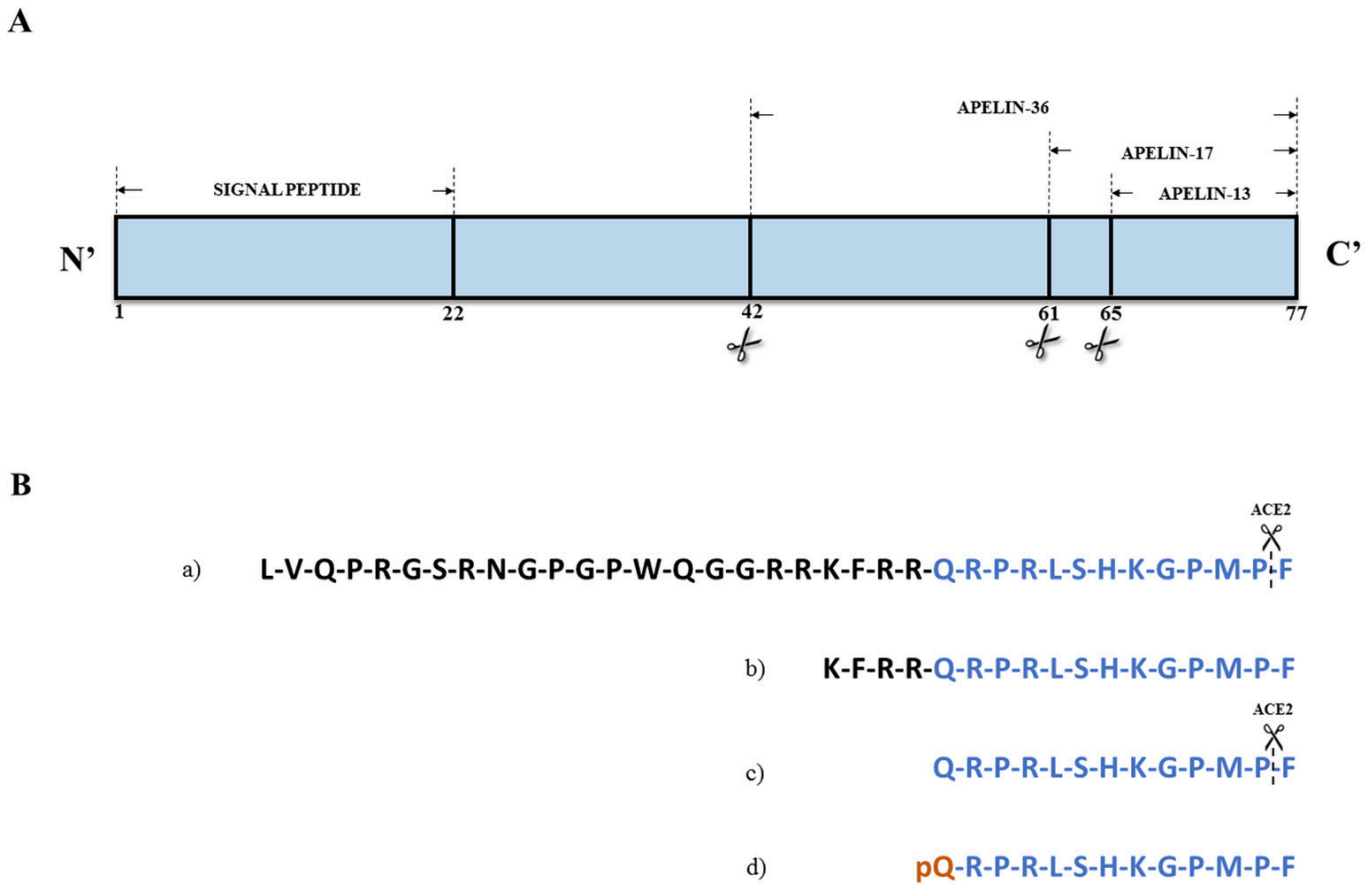
**(A)** Structure of apelin precursor – 77-amino acids preproapelin. **(B)** Amino acid sequences of (a) apelin-36, (b) apelin-17, (c) apelin-13, and (d) [Pyr1]apelin-13. Angiotensin converting enzyme 2 (ACE-2) can hydrolase apelin-13 and apelin-36 removing C-terminal residue. Based on Habata et al. (1999).

Apelin, an endogenous peptide, was identified as a ligand of the orphan G protein-coupled receptor APJ, so the name apelin comes from APJ Endogenous Ligand. Apelin was first isolated from the bovine stomach (Tatemoto et al., 1998). The APJ human gene (*APLNR*) encodes a seven-transmembrane protein closely related to the angiotensin receptor (O'Dowd et al., 1993). Both proteins share an identity of 54% in the transmembrane regions. However, angiotensin II does not bind to APJ (Lee et al., 2000). In addition to angiotensin II, apelin is also a substrate for catalytic angiotensin-converting enzyme 2 (ACE2) activity *in vitro* (Sato et al., 2013). The apelin receptor contains consensus sites for palmitoylation, glycosylation, and phosphorylation by cyclic adenosine monophosphate (cAMP)-dependent protein kinase (O'Dowd et al., 1993; Tatemoto et al., 1998). The apelin-encoding gene (*APLN*) is located on chromosome Xq25-26.1 (Lee et al., 2000) and encodes a 77-amino acid prepropeptide (Figure 1A). Preproapelin is cleaved from its C-terminus to produce a mature apelin peptide, apelin-36, or a family of shorter peptides (apelin-17,−12, and−13), the latter of which also exists as a pyroglutamyl form, [Pyr^1^]apelin-13 (Habata et al., 1999) (Figure 1B).

In 1999, Habata et al. demonstrated the secretion of large amounts of apelin peptides into bovine colostrum and milk (Habata et al., 1999). Tissue expression analyses revealed, that the distribution of preproapelin and APJ mRNA were similar. The apelin peptide was detected in the spinal cord and several human brain regions (Matsumoto et al., 1996; Edinger et al., 1998; Lee et al., 2000). In rats, preproapelin and APJ mRNA were detected in brain and peripheral tissues, including testis, intestine, kidney, and in the fetus (Lee et al., 2000; O'Carroll et al., 2000). High level apelin mRNA levels were also identified in the rat heart (Lee et al., 2000), lung, and mammary glands (Kawamata et al., 2001), however only faint signals were detected in spleen and liver (Lee et al., 2000). Tissue expression of apelin-17 in humans was also determined using immunohistochemistry; apelin-17 is highly expressed in chondrocytes, endothelial cells, the heart, skin, brain, spleen, thymus, and lungs. An intermediate expression level of apelin was found in skeletal muscle. In the liver, pancreas, and kidney, apelin-17 was detected in relatively low levels (De Falco et al., 2002). Apelin was also highly expressed in adipocytes. As a hormone released by adipose tissue, apelin belongs to adipokines family. Adipokines display many properties, such as pro-inflammatory classical cytokines (tumor necrosis factor alpha [TNF-α], interleukin-6 [IL-6]), chemokines (monocyte chemoattractant protein-1), proteins involved in vascular homeostasis (plasminogen activator inhibitor), regulation of blood pressure (angiotensinogen), glucose homeostasis (adiponectin), lipid metabolism (retinol binding protein), and angiogenesis [vascular endothelial growth factor [VEGF](Trayhurn et al., 2006)].

Its extensive tissue distribution suggests, that the apelin/APJ system, also known as an apelinergic system, might be involved in many physiological processes, such as regulation of body fluid homeostasis (Reaux et al., 2001), blood pressure (Tatemoto et al., 2001), endocrine stress response (Taheri et al., 2002; O'Carroll et al., 2003), cardiac contractility (Szokodi et al., 2002), angiogenesis (Zhang et al., 2016), and energy metabolism (Bertrand et al., 2015). Additionally, apelin participates in pathological processes, including heart failure (Földes et al., 2003), obesity (Boucher et al., 2005), diabetes (Li et al., 2006), and cancer (Wang et al., 2008).

## Apelin in cardiovascular diseases

Under normal conditions apelin and APJ are expressed in cardiac myocytes. Apelin has a positive inotropic effect *in vitro* (Szokodi et al., 2002) and is involved in lowering arterial blood pressure (Tatemoto et al., 2001), inducing arterial vasodilation (Japp et al., 2008), and improvement of cardiac output (Japp et al., 2010). The first connection between apelin and pathology of the cardiac system was made in 2003. Földes et al. demonstrated higher expression levels of apelin mRNA in failing human hearts in compared to normal tissue, and suggested, that apelin might be involved in the pathophysiology of human heart failure (Földes et al., 2003). Apelin increases cardiac output and lowers blood pressure and peripheral vascular resistance in patients with heart failure (Japp et al., 2010). Moreover, [Pyr^1^]apelin-13 injection into a rat model of myocardial infarction resulted in decreased infarct size, and increased heart rate and serum nitric oxide level in consecutive days, indicating that apelin has a sustained cardioprotective effect against myocardial infarction (Azizi et al., 2013). Another study revealed the connection between dramatic improvement of cardiac function and significant upregulation of stromal cell-derived factor 1/C-X-C chemokine receptor type 4 expression in postmyocardial infarction mice after administration of apelin-13 (Li et al., 2012). This peptide can also abolish reactive oxygen species (ROS) formation, reduce oxidative stress and prevent cardiac hypertrophy (Foussal et al., 2010). Interestingly, the loss of apelin in apelin-knockout mice increased myocardial infarction mortality, infarct size, and inflammation, with a reduction of the prosurvival pathway *via* phosphatidyl inositol 3-kinase/protein kinase B (PI3K/Akt) (Wang et al., 2013).

Hypertension is a cardiovascular condition, characterized by increased arterial blood pressure. Long-term high blood pressure could be a risk factor for many cardiovascular events, such as coronary artery disease, stroke, ischemic heart disease, myocardial infarction, and peripheral vascular disease (Alam et al., 2017). Apelin and APJ mRNA expression levels were reduced in rats with hypertension (Akcilar et al., 2013), which correlates with the significantly lower level of plasma apelin in the group of newly diagnosed hypertensive patients (Sonmez et al., 2010). APJ could be responsible for the action of apelin on regulating blood pressure. The protein expression level of this receptor was decreased in myocytes isolated from the left ventricular tissue of hypertensive rats with heart failure, compared to the control group. After the exogenous infusion of [Pyr1]-apelin-13, APJ levels were significantly increased. Reduced left ventricular systolic pressure was observed in hypertensive rats after apelin administration (Pang et al., 2014). Multiple findings suggest, that the hypotensive effect of apelin and APJ receptor might be mediated through nitric-oxide synthase (eNOS) (Ishida et al., 2004). Studies examining olmesartan, an angiotensin II receptor antagonist, as a treatment for hypertension in rats with heart failure, also found, that apelin can be involved in regulation of the Akt/eNOS pathway. (Fukushima et al., 2010). The APJ receptor can also act as a pressure sensor to respond to cardiac hypertrophy. In H9c2 rat myocardial cells, static pressure (180 mmHg) increased the expression of APJ protein, activated the PI3K/Akt pathway, induced cell autophagy, and stimulated myocardial hypertrophy (Xie et al., 2014).

Several studies have demonstrated that apelin can be treated as a biomarker of cardiovascular diseases. In patients with coronary artery disease, serum apelin-12 levels were reduced (Kadoglou et al., 2010). Decreased myocardial (Chandrasekaran et al., 2010) and serum (Ye et al., 2015) apelin-12 production was also characteristic for patients with systolic left ventricular dysfunction disease. The secretion pattern of apelin-12, measured as a concentration of this peptide in the coronary sinus, aorta, and renal vein, differed to that of brain natriuretic peptide, an internal control exclusively produced in heart (Chandrasekaran et al., 2010). These findings were also verified by Helske et al., who demonstrated altered transcardiac arteriovenous gradients of circulating in serum apelin-12 in response to left ventricular pressure overload (Helske et al., 2010). The level of apelin-36 was measured in patients and rats with left ventricular hypertrophy. In both groups, while the expression of myocardial apelin decreased, apelin plasma levels increased. The correlation between plasma apelin and left ventricular mass index in human and rats demonstrated, that this peptide might be used as a biomarker of left ventricular hypertrophy (Falcão-Pires et al., 2010). Meta-analysis of data indicated, that serum apelin (all forms) might be a prominent athero-protective marker against the development of coronary artery diseases (Chen et al., 2017b). The expression changes of apelin/APJ system in cardiovascular diseases are shown in Table 1.

**Table 1:** Expression of apelin/APJ in cardiovascular diseases, and organs under hypoxia.

**Disease**	**Patient/tissue/cell line**	**mRNA**	**Protein**	**References**
Failing heart	Human idiopathic dilated cardiomyopathy tissue	*APLN* ↑	–	Földes et al., 2003
Hypertension	Human plasma	–	Apelin-12 ↓	Sonmez et al., 2010
	Rat plasma	*APLN* ↓ *APLNR* ↓	–	Akcilar et al., 2013
	Rat left ventricular myocytes	–	APJ ↓	Pang et al., 2014
Coronary artery disease	Human plasma	–	Apelin-12 ↓	Kadoglou et al., 2010
Left ventricular hypertrophy	Human left ventricular myocytes	–	Apelin-12 ↓	Chandrasekaran et al., 2010
	Human plasma	–	Apelin-12 ↓	Ye et al., 2015
	Rat left ventricular myocytes	–	Apelin-36 ↓	Falcão-Pires et al., 2010
	Rat plasma	–	Apelin-36 ↑	
Retinal ischemia	Rat retinal Müller cells	*APLN* ↑ *APLNR* ↑	Apelin ↑ (unspecified) APJ ↑	Wang et al., 2012
Heart ischemia	Rat hearts	–	APJ ↑	Rastaldo et al., 2011
Brain ischemia	Rat and mouse hippocampus	–	APJ ↓	Fan et al., 2017
Pulmonary hypertension	Human plasma	–	Apelin-12 ↓	Chandra et al., 2011
	Mouse lung	*APLN* ↑ *APLNR* ↑	–	
Adipocyte hypoxia	Human adipocytes	APLN ↑	–	Kunduzova et al., 2008; Geiger et al., 2011
	Human adipocytes medium	–	Apelin-13 ↑ Apelin-36 ↑	Geiger et al., 2011
Ischemic retinopathy	Mouse retinas	*APLN* ↑ *APLNR* ↑	APJ ↑	Kasai et al., 2010
Portal hypertension	Rat mesentery, intestine, portal vein, and mesenteric artery	*APLN* ↑ *APLNR* ↑	–	Tiani et al., 2009

*↑, increase; ↓, decrease; –, not clear*.

All of these results, suggest that apelin could be responsible for increased cardiac output and cardioprotective effect against myocardial infarction and oxidative stress. Furthermore, the apelinergic system can play an important role in the regulation of blood pressure, acting as a pressure sensor to respond to cardiac hypertrophy. The hypotensive effect of apelin could be mediated through the Akt/eNOS pathway. The role of the apelin/APJ system in cardiac hypertrophy was well summarized by Lu et al. (2017). Additionally, in patients with different cardiovascular diseases, such as coronary artery disease, systolic left ventricular dysfunction disease, and left ventricular hypertrophy, apelin concentration is also altered, suggesting that apelin peptides could be successfully used as a biomarker of cardiovascular system pathologies.

## Apelin in hypoxia

Hypoxia is a condition of the body often caused by interrupted blood flow, inflammation, sepsis or hypertension, leading to the release of hypoxia-inducible factor (HIF-1). This short-lived macromolecule is a transcription factor that modifies and regulates cell metabolism to increase or decrease oxygen concentration. Sustained hypoxia causes organ and tissue damage (Pozo Devoto et al., 2013). This condition enhances the expression of HIF-1, leading to upregulation of apelin/APJ signaling and activation of PI3K/Akt and extracellular signal-regulated kinase pathways (ERK) (Zhang et al., 2015a,b). The expression changes of the apelin/APJ system in organs under hypoxia are shown in Table 1.

The main reasons for hypoxia are ischemia and reperfusion (I/R)—pathological conditions characterized by restriction of blood delivery to organs and tissues. I/R contribute to a wide range of pathologies, including myocardial infarction, ischemic stroke, acute kidney injury, trauma, circulatory arrest, sickle cell disease, and sleep apnea (Eltzschig and Eckle, 2011). Renal I/R can occur as a consequence of systemic hypotension, cardiac arrest, renovascular surgery, and aortic clamping. Various pharmacological methods have been investigated for the treatment of renal I/R injury (He et al., 2015). Sagiroglu et al. examined the effect of apelin on renal functions following renal I/R. In that study, apelin-13 administrated pre-operatively to a I/R rat model resulted in protective, functional, and histopathological effects of renal I/R injury (Sagiroglu et al., 2012). The same conclusions have been drawn in another study, indicating that apelin-13 applied after rat kidney I/R injury increased antioxidant enzyme activity in a dose-dependent manner, prevented lipid oxidation, and improved renal functions (Bircan et al., 2016). Incubation of rat primary Müller cells with apelin-13 caused increased cell viability, migration, and expression of glial fibrillary acidic protein and VEGF. This, in turn, led to the significant protection of Müller cells against hypoxia conditions (Wang et al., 2012; Lu et al., 2013).

Apelin is also able to protect the heart against I/R injury both *in vivo* and *in vitro*. When administrated immediately after ischemia to isolated perfused rat hearts, apelin-13 protected the heart, limiting infarct size and improving postischemic mechanical recovery (Rastaldo et al., 2011). On the other hand, administration of apelin-12 to rats before ischemia or at the beginning of reperfusion reduced I/R injury. This cardioprotective effect compromises prevention and attenuation of oxidative stress by increasing the activity of antioxidant enzymes in postischemic hearts. This leads to inhibition of lipid peroxidation and reduced ROS formation (Pisarenko et al., 2011, 2014; Pisarenko O. et al., 2015). A structural analog of apelin-12-modified apelin-12 (MA)- injected into isolated perfused rat hearts, also reduced cardiomyocyte damage and improved cardiac dysfunction. This cardioprotective effect was mediated by protein kinase C (PKC), PI3K, and MAPK/ERK kinase 1/2 signaling (Pisarenko O. I. et al., 2015). Several signal transduction pathways -PI3K/Akt, ERK, mitogen-activated protein kinase (MAPK), and eNOS—have been proposed as the mechanism underlying the protective effect of the apelinergic system. Each of these signaling pathways is involved in protection against I/R injury, especially by the modulation of endoplasmic reticulum stress-induced apoptotic activation during the first 24 h of reperfusion (Tao et al., 2011).

The apelinergic system is involved in cerebral ischemia. Apelin-13 reduced brain infarct size in a dose-dependent manner in a transient model of focal stroke in rats. The central applications of this peptide showed a protective effect against cerebral damage and brain edema, thus preventing apoptosis (Khaksari et al., 2012). Additionally, apelin-13 had an anxiolytic effect on rats exposed to chronic normobaric hypoxia. These rats exhibited anxiety-like behavior, which might be associated with inhibition of nuclear factor kappa-light-chain-enhancer of activated B cells (NF-κB) activation in microglial of the hippocampus (Fan et al., 2017). This peptide is also involved in protecting the blood-brain barrier from ischemic injury. Administration of apelin-13 in a mouse model with middle cerebral artery occlusion resulted in reduced infarct volume. The authors indicated that this is connected with aquaporin-4 upregulation, which could be a result of the activation of the ERK and PI3K/Akt pathways (Chu et al., 2017). In the same experimental model, apelin-36 injection reduced infarct volume and neuronal apoptosis (Gu et al., 2013). The same conclusions were reached by Yang et al., demonstrating the neuroprotective effect of apelin-13 against I/R through PI3K/Akt and ERK pathways using a cerebral I/R mouse model (Yang Y. et al., 2014). Apelin-13 can also inhibit apoptosis of neuronal cells in I/R cerebral mice through inhibition of immunoreactivity of pro-apoptotic factors and promotion of immunoreactivity of anti-apoptotic factors (Yan et al., 2015). AMP-activated protein kinase (AMPK) could be the mediator of the anti-apoptotic protection of apelin, since apelin-13 upregulated its level after cerebral I/R in mice (Yang et al., 2016b).

In pulmonary hypertension, the level of pulmonary tissue and plasma apelin (all forms) was unchanged by hypoxia. Moreover, in arteries of normoxic rats, apelin modulated vasoconstrictor tone, which was not observed in hypoxic animals. However, the level of apelin in the right ventricle was related to right ventricular pressure, suggesting that apelin could be used as a pulmonary hypertension marker (Andersen et al., 2009). Exacerbation of pulmonary hypertension induced by hypoxia was observed in mice, and this effect was mediated by downregulation of eNOS. Moreover, the critical mediators of apelin-APJ signaling in pulmonary artery endothelial cells were AMPK and Kruppel-like factor 2. Reduced apelin-12 levels were also observed in patients with pulmonary hypertension, suggesting its importance in this disease (Chandra et al., 2011). Interestingly, *in vitro* studies on pulmonary arterial smooth muscle cells (PASMCs) have indicated that hypoxia increases proliferation and migration of these cells. PASMCs proliferation was related to activation of autophagy in response to hypoxia. Apelin treatment under hypoxic conditions resulted in a decrease in PASMCs proliferation and migration through inhibition of autophagy, regulated by activation of the PI3K/Akt/mechanistic target of the rapamycin (mTOR) pathway (Zhang et al., 2014).

In conditions combining obesity and cardiac I/R injury, apelin-13 administration to mice decreased myocardial expression of pro-apoptotic B-cell lymphoma 2 (Bcl-2)-associated X protein and increased the expression of anti-apoptotic Bcl-2, leading to reduced myocardial apoptosis. Inhibition of apoptotic cell death was associated with a reduction of hypoxia-induced ROS production and attenuation of oxidative stress through the forkhead box protein O1 pathway (Boal et al., 2016).

Another metabolic disease, diabetes mellitus, exerts metabolic changes in erythrocytes, leading to oxidative stress. In diabetic rats, erythrocytes deformability was altered in myocardial I/R injury. However, apelin-13 administration before ischemia had a protective effect against these perturbations (Kartal et al., 2017). Hypoxia could also induce apelin secretion in human adipocytes. The level of secreted apelin-13 and apelin-36 was increased in adipocytes, and HIF-1 was the major factor involved in this process (Geiger et al., 2011). Moreover, apelin has an antioxidant effect in adipocytes. Apelin peptide treatment decreased ROS production and increased activity of antioxidant enzymes, such as Cu/Zn superoxide dismutase, catalase, and glutathione peroxidase. Apelin could also attenuate the ROS-stimulated release of pro-inflammatory adipocytokines and free fatty acids (Than et al., 2014). Furthermore, in adipocytes after hypoxic exposure, HIF-1 was responsible for upregulation of apelin level in response to insulin (Glassford et al., 2007). Adipocyte-released apelin upregulated by hypoxia could play a critical role in the development of the functional vascular network in adipose tissue (Kunduzova et al., 2008).

To summarize, apelin plays a protective role against ischemia through the PI3K/Akt, ERK, MAPK, AMPK, and eNOS pathways. Apelin is also responsible for decreased ROS formation and increased activity of antioxidant enzymes in adipocytes. Apelin secreted by adipocytes could play an important role in vascular network development in adipose tissue.

## Apelin in angiogenesis

Angiogenesis is the physiological process of forming new blood vessels from existing vessels. This process is crucial for supplying tissues with oxygen and nutrients and for removing metabolites, such as carbon dioxide. Prolonged angiogenesis often indicates a pathological condition, such as arthritis, diabetic retinopathy or cancer progression (Al-Abd et al., 2017).

In ischemic disorders, HIFs transcription factors are upregulated, leading to the alternation of the expression of the angiogenesis-related factors gene expression. The proliferation of mesenchymal stem cells and upregulation of HIF-1 expression could be mediated through the apelin-APJ/autophagy pathway (Li et al., 2015). Administration of apelin also promoted mesenchymal stem cells survival and vascularization under hypoxic-ischemic conditions. This process might be connected with upregulation of VEGF (Hou et al., 2017).

Apelin signaling is also essential for angiogenesis promotion during portal hypertension (Tiani et al., 2009). Furthermore, administration of apelin-13 to rats with ischemic stroke led to increased forming of new blood vessels (Chen D. et al., 2015). Hypoxia conditions might also induce the expression of adipocyte-derived apelin, which could have important consequences for the relationship between adipose tissue and endothelial vascular cell function in the control of angiogenesis (Kunduzova et al., 2008). One of the suggested mechanisms underlying the role of apelin in angiogenesis is activation of AMPK and PI3K/Akt signaling. Moreover, eNOS, an important mediator in angiogenesis, is activated through direct phosphorylation by AMPK and Akt (Yang X. et al., 2014; Zhang et al., 2016).

The apelin/APJ system might be involved in pathological angiogenesis (Wu et al., 2017). In a mouse model of oxygen-induced retinopathy, the expression of apelin was dramatically increased during hypoxia and was significantly higher than the expression of VEGF. Moreover, APJ was highly expressed in proliferative capillary endothelial cells. Additionally, the suppression of apelin expression in apelin-knockout mice led to a limited proliferation of endothelial cells but induced retinal vessel maturation by promoting pericyte recruitment (Kasai et al., 2010, 2013).

All of these results indicate that apelin might mediate angiogenesis by upregulation of HIF-1, VEGF, and VEGFR (vascular endothelial growth factor receptor 2), as well as by activation of the AMPK/eNOS and PI3K/Akt/eNOS pathways. During pathological retinal angiogenesis, the expression of apelin and APJ is also increased.

## Apelin in obesity

Adipokines are biologically active molecules secreted by adipose tissue, the complex organ, in which adipocytes are the main cellular component (Tapan et al., 2010). In addition to adipocytes, this dynamic tissue is also composed of stromal-vascular fraction, compromising blood cells, pericytes, endothelial cells, and adipose precursors. Adipose tissue does not only perform a fat storage function; it is also synthesizing some biologically active compounds, which regulate metabolic homeostasis (Coelho et al., 2013). Apelin, as a member of the adipose tissue-derived peptides, might contribute to obesity-related disorders. However, its role remains unclear and experimental findings have been inconsistent. In the studies examining obese young patients, the apelin-12 plasma level was decreased, relative to healthy patients, which could be associated with severity of insulin resistance and adiposity (Tapan et al., 2010; Kotanidou et al., 2015). However, another study found that its plasma level was significantly increased in obese female children in comparison to non-obese children (Ba et al., 2014). Another study reported increased apelin-12 serum concentration in patients with obesity and obesity-related insulin resistance, which could be caused by impaired insulin sensitivity (Krist et al., 2013). These opposite findings might be explained by differential expression of apelin across tissues. In the obese and insulin-resistant high-fat diet female mice, the plasma apelin-12 concentration was not altered, but the level of apelin gene-expression was elevated in white-adipose tissue and reduced in brown-adipose tissue, liver, and kidneys, suggesting that the apelinergic system could be implicated in several dysfunctions in these tissues under obesity (Butruille et al., 2013). Additionally, in obese patients after hypocaloric diet-induced weight loss, the plasma apelin (all forms) level was significantly decreased. The diet-induced changes in plasma apelin levels directly correlated with the diet-induced decrease of metabolic variables, such as plasma insulin and TNF-α levels. However, expression of apelin and APJ was also decreased after low-calorie diet (Castan-Laurell et al., 2008).

Some data have indicated that there is a correlation between plasma insulin level and apelin expression in adipocytes. Administration of insulin into obese mice increased apelin gene transcription, what could be associated with activation of the PI3K/Akt, PKC, and MAPK pathways (Boucher et al., 2005). Apelin is also involved in a decrease of lipolysis in adipocytes. Administration of [Pyr1]-apelin-13 inhibited isoproterenol-induced lipolysis in cultured adipocytes through two possible mechanisms: attenuating PKA-mediated or increasing AMPK-mediated Ser-563 hormone-sensitive lipase phosphorylation (Yue et al., 2011). Another research revealed, that [Pyr1]-apelin-13 inhibited adipogenesis of pre-adipocytes by the AMPK and MAPK/ERK pathways. Moreover, exogenous apelin decreased the number of differentiated adipocytes and increased the size of lipid droplets inside the cells, suggesting that apelin might suppress lypolysis (Than et al., 2012).

Interestingly, adipose tissue growth is correlated with angiogenesis. The accumulation of adipocytes occurring under obesity could be closely linked with the structure and function of lymphatic vessels. Apelin signaling leads to enhanced lymphatic and blood vessels integrity. Increased permeability of lymphatic and blood vessels induced by dietary fatty acids, which leads to a block of fat accumulation was inhibited by apelin (Sawane et al., 2013).

These results hint that apelin might play an important role in obesity. The plasma apelin level is changed in obese patients compared to non-obese controls. Apelin inhibits lipolysis in adipocytes and is involved in angiogenesis in adipose tissue. However, the findings of studies investigating the role of apelin in obesity are inconsistent, and there are still many gaps in this topic. The expression changes of the apelin/APJ system under obesity are shown in Table 2.

**Table 2:** Expression of apelin/APJ in obesity, diabetes mellitus, and diabetes-related diseases.

**Disease**	**Patient/tissue/cell line**	**mRNA**	**Protein**	**References**
Obesity	Human plasma	–	Apelin-12 ↓	Tapan et al., 2010; Krist et al., 2013; Kotanidou et al., 2015
		–	Apelin-12 ↑	Ba et al., 2014
Diabetes mellitus	Human plasma	–	Apelin-12 ↑	Habchi et al., 2014; Ma et al., 2014
	Rat heart	–	Apelin ↑ (unspecified)	Akcilar et al., 2015
Diabetic retinopathy	Human vitreous body	–	Apelin-13 ↑	Tao et al., 2010
Diabetic nephropathy	Mouse kidney cortex	–	Apelin-13 ↓	Day et al., 2013
Diabetic cardiomyopathy	Mouse heart	–	Apelin ↑ (unspecified)	Zeng et al., 2014

*↑, increase; ↓, decrease; –, not clear*.

## Apelin in a type 2 diabetes

Apelin is expressed in human, mouse, rat, pig and cat pancreatic islets and is regulated by glucocorticoids, but not by glucose (Ringström et al., 2010). Apelin plays a beneficial role in energy metabolism by increasing glucose uptake and insulin sensitivity (Bertrand et al., 2015). However, many controversies surround the relationship between apelin signaling, insulin sensitivity, and glucose uptake, which are discussed in the review by Xu et al. (2011). In diabetic patients apelin-12 concertation was increased (Habchi et al., 2014), extending susceptibility to diabetes (Zheng et al., 2016), thus, examining plasma apelin level could be used as a method to predict the development of type 2 diabetes (Ma et al., 2014; Hu et al., 2016). Furthermore, an augmented level of apelin in rats suggests, that it has a strong anti-type 2 diabetic activity and acts as an insulin-sensitizing agent (Akcilar et al., 2015).

Administration of apelin-13 to mice results in an eNOS-dependent decrease in glycemia and stimulation of glucose turnover. Moreover, AMPK signaling was a potential upstream target of eNOS-mediated stimulation of glucose transport (Dray et al., 2008). Additionally, [Pyr1]-apelin-13 increased glucose uptake in adipocytes by inducing translocation of glucose transporter type 4 (GLUT-4) in a PI3K/Akt-dependent manner and mediated inflammatory response in insulin-resistant adipocytes (Zhu et al., 2011). Apelin could also be involved in regulation of blood glucose level by AMPK activation and cAMP decrease (Alipour et al., 2017).

In diabetes-related diseases, such as diabetic retinopathy or nephropathy, the apelin-13 level was significantly elevated in comparison to non-diabetic organs. Furthermore, in the kidneys diabetic rats, administration of apelin-13 restored the downregulated expression of the antioxidant enzyme catalase, suggesting a renoprotective effect of apelin through antioxidant pathways (Day et al., 2013). Apelin was also responsible for podocyte apoptosis, which was negatively correlated with podocyte autophagy in diabetic mice with nephropathy. Moreover, the mTOR pathway has been proposed as the mechanism responsible for inhibition of podocyte autophagy by apelin. Additionally, apelin-13 might play a role in retinal neovascularization under diabetic retinopathy (Tao et al., 2010; Day et al., 2013; Liu et al., 2017). In mice with diabetic cardiomyopathy, adenoviral administration of the apelin gene led to increased expression of VEGF/VEGFR2 and angiopoietin-1/tyrosine-protein kinase receptor. Overexpression of apelin resulted in augmented myocardial angiogenesis, attenuated diabetic cardiac hypertrophy, and improved cardiac function (Zeng et al., 2014; Hou et al., 2015). There is also a connection between type 2 diabetes and cancer. A positive correlation was observed *in vitro* in breast, colon, and pancreas cancers. Hyperinsulinemia is likely the major factor, that plays a role in these associations. Insulin resistance, which leads to hyperinsulinemia, might serve as a potential target for cancer therapy (Cannata et al., 2010). The expression changes of the apelin/APJ system under diabetes mellitus and diabetes-related diseases are shown in Table 2.

All these findings indicate that the stimulation of glucose uptake by apelin is possible through translocation of GLUT-4 in a PI3K/Akt-dependent manner. In this process, the AMPK/eNOS pathways are also involved. In diabetes-related diseases, such as retinopathy, nephropathy or cardiomyopathy apelin has a protective effect against oxidative stress and apoptosis through the mTOR pathway.

## Apelin in cancer

### Role of apelin in different types of cancer

There are several well-known hallmarks of cancer, including sustaining proliferative signaling, evading growth suppression, activating invasion and metastasis, enabling replicative immortality, inducing angiogenesis, and resisting cell death (Hanahan and Weinberg, 2011).

Apelin might be involved in the regulation of tumor growth, cancer cell migration, neoangiogenesis, apoptosis suppression, and even metastasis induction. Various apelin peptides can stimulate tumor growth and proliferation of many types of cancer cells, including cholangiocarcinoma (CAA) (Hall et al., 2017), non-small cell lung cancer (NSCLC) (Berta et al., 2010), gastric cancer (Feng et al., 2016), prostate cancer (Tekin et al., 2014), ovarian cancer (Hoffmann et al., 2017), and oral squamous cell carcinoma (Heo et al., 2012). The expression changes of the apelin/APJ system in cancer are shown in Table 3.

**Table 3:** Expression of apelin/APJ in different types of cancer.

**Disease**	**Patient/tissue/cell line**	**mRNA**	**Protein**	**References**
Lung cancer	Non-small cell lung carcinoma	*APLN* ↑	–	Berta et al., 2010
	Adenocarcinoma	–	APJ ↑	Yang L. et al., 2014
Cholangiocarcinoma	Cholangiocarcinoma	*APLN* ↑ *APLNR* ↑	–	Hall et al., 2017
	Cholangiocarcinoma cell lines	–	Apelin-36 ↑ APJ ↑	
Liver cancer	Hepatocellular carcinoma	*APLN* ↑	–	Muto et al., 2014
Gastric cancer	Gastroesophageal Cell Carcinoma	–	Apelin ↑ (unspecified)	Diakowska et al., 2014; Feng et al., 2016
	Plasma	–	Apelin ↑ (unspecified)	Diakowska et al., 2014
	Gastric cancer	–	APJ ↑	Hao et al., 2017
	Adenomas and adenocarcinomas	–	Preproapelin ↑ APJ ↑	Picault et al., 2014
	Colon cancer cell lines	–	Preproapelin ↑ APJ ↑	
	Colon carcinoma	*APLNR ↑*	–	Chen et al., 2017a
Prostate cancer	Prostate cancer	*APLN* ↑	–	Wan et al., 2015
Ovarian cancer	Ovarian cancer	*APLN* ↑ *APLNR* ↑	–	Hoffmann et al., 2017
Breast cancer	Plasma	–	Apelin-36 ↑	Salman et al., 2016
Renal cancer	Clear renal cell carcinoma	*APLN*_◦_	–	Zhang et al., 2017
Squamous cell carcinoma	Oral squamous cell carcinoma	–	Apelin-36 °	Heo et al., 2012
Multiple myeloma	Plasma	–	Apelin ↑ (unspecified)	Maden et al., 2017
Glioblastoma	Glioblastoma	*APLN* ↑	–	Harford-Wright et al., 2017
Obesity-related colon cancer	Human plasma	–	Apelin-12 ↑	Al-harithy and Al-otaibi, 2015
Obesity-related endometrial cancer	Human plasma	–	Apelin-36 ↑	Altinkaya et al., 2015; Salman et al., 2016

*↑, increase; ↓, decrease; °, positive result; –, not clear*.

The apelin/APJ system is involved in the induction of cell migration. A pyroglutamyl form of apelin [Pyr1]-apelin-13 could stimulate the migration of human embryonic kidney cells with APJ overexpression. This peptide activated phosphorylation of Akt and focal adhesion kinase (FAK), which was mediated by the PI3K signaling pathway (Hashimoto et al., 2005). Apelin also increased the migratory abilities of human lung adenocarcinoma (Lv et al., 2016), gastric cancer (Feng et al., 2016) and oral squamous cell carcinoma (Heo et al., 2012). There are several possible mechanisms leading to the regulation of cell migration, one of which is the p-21 activated kinase (PAK1)/cofilin signaling pathway. Cofilin is a member of an actin-binding protein family involved in cell migration by the organization of actin filaments. Cofilin can be activated by PAK1 kinase through indirect interaction. Other mediators of this signaling pathway remain unknown (Lv et al., 2016). It is also possible, that apelin induces migration *via* MAPK/ERK in oral cancer cells (Heo et al., 2012), AMPK, PI3K/Akt, and peroxisome proliferator-activated receptor (PPAR) pathways in ovarian cancer cells (Dupont et al., 2012).

Migration of cancer cells is strictly associated with metastasis. In mice, apelin-13 could stimulate lymph nodes metastasis of implanted apelin-overexpressing melanoma cells (Berta et al., 2014). It is also known that epithelial-mesenchymal transition (EMT) can be involved in the initiating stage of cancer cell metastasis. The role of the apelinergic system in EMT remains controversial. Although apelin was able to induce cell migration and metastasis in primary human renal proximal tubular epithelial cells, apelin-13 also inhibited TGF-β (transforming growth factor-β) -induced EMT. This inhibitory effect contributed Smad-2/3 and PKC-ε (Wang et al., 2017).

Tumor hypoxia is one of the main pathological factors that contributes to shift of the angiogenic balance to pro-angiogenic conditions. The release of pro-angiogenic factors from tumor and host cells, like macrophages, also causes perturbation in the vascular network. These pro-angiogenic factors can work as a chemotactic signal resulting in migration and proliferation of endothelial cells within the tumor tissue, and formation of the new vascular networks (Al-Abd et al., 2017). This process could be associated with the activation of several signaling pathways, including PI3K, eNOS, and phosphorylation of FAK (Lamalice et al., 2007). The apelinergic system might be involved in angiogenesis in tumor progression. Implantation of apelin-overexpressing NSCLC cells into mice resulted in accelerated tumor growth *in vivo* with increased microvessel density (MVD) (Berta et al., 2010). In patients with hepatocellular carcinoma (HCC), MVD was higher than in non-tumor tissue. The transcript levels of apelin and pro-angiogenic factors (angiopoietin 1 [ang-1]), angiopoietin 2 [ang-2]) were also elevated in the same model. In the HCC tumor mouse model, the APJ antagonist, F13A, inhibits tumor growth, suggesting, that the apelinergic system stimulates tumor growth (Muto et al., 2014). Additionally, in hepatocellular cell lines, upregulation of the *APLN* gene was identified as promoting angiogenesis, invasion, and metastasis of cancer cells (Lin and Chuang, 2013). Moreover, the Matrigel tube formation was used to show that apelin-13 effectively stimulates differentiation lymphatic endothelial cells (LEC) into vascular structures *in vitro*. Furthermore, implantation of melanoma cells overexpressing apelin into mice resulted in an increase of intratumor lymphangiogenesis and metastasis to lymph nodes (Berta et al., 2014). In human CAA cell lines [Pyr1]-Apelin-13 stimulated cell proliferation and increased expression of angiogenesis factors, such as VEGF-A VEGF-C, ang-1, and ang-2, whereas the APJ antagonist, ML221, has an opposite effect, suggesting that [Pyr1]-apelin-13 could be a stimulator of proliferation and angiogenesis of CAA cells (Hall et al., 2017).

The protective effect of apelin against apoptosis is also described. In primary rat pericyte cells apelin-13 increased the viability of the cells under hypoxic conditions. This peptide significantly decreased the level of caspase-3 activity, which is crucial apoptosis mediator (Chen L. et al., 2015). In vascular smooth muscle cells apelin-13 protected cells against serum deprivation-induced apoptosis by the PI3K/Akt pathway (Cui et al., 2010). Apelin-derived peptides- [Pyr1]-apelin-13, apelin-13, and apelin 36, protected colorectal cancer cells from apoptosis induced by pro-apoptotic factors. Apelin fragments also decreased caspase-3 activity and poly-ADP ribose polymerase protein proteolysis, suggesting that apelin is involved in resisting cell death during cancer progression (Picault et al., 2014).

Many expression changes of the apelin/APJ system were observed in various types of cancer (Yang et al., 2016a). In NSCLC the level of apelin mRNA and apelin-36 peptide were significantly higher in the patient's tumor samples compared to normal tissue. In the group of advanced NSCLC patients a significant correlation between apelin-12 level and overall survival was detected. However, there was no association with differential treatment response rates, different chemotherapy regiments or hematological side effects (Ermin et al., 2016). Another study revealed, that the expression of APJ protein was higher in lung adenocarcinoma tissue, than in submucous bronchial tissue. The level of plasma apelin-13 was also significantly higher in patients with this type of cancer. *In vitro* studies have demonstrated, that apelin-13 promotes the proliferation of human lung adenocarcinoma cells through an upregulation of cyclin D1 level and thus accelerating the conversion of G0/G1 to S phase in the cell cycle. Furthermore, apelin-13 induced ERK1/2 phosphorylation and autophagy inhibition, detected as an increase in microtubule-associated protein 1 light chain 3 alpha and beclin 1 levels, which both led to a proliferation of cancer cells (Yang L. et al., 2014). Interestingly, apelin-13 was able to impact cell migration. Cell migration assays revealed, that apelin-13 and APJ were responsible for increased migration abilities of human lung adenocarcinoma cells *via* the PAK1-cofilin signaling pathway (Lv et al., 2016).

In gastroesophageal cell carcinoma, the apelin serum and tissue levels are significantly higher than in healthy samples (Diakowska et al., 2014). In gastric cancer (GC) tissue, the apelin level was closely associated with clinical features and prognosis in GC patients. Patients with high tumor apelin expression had a shorter overall survival period than those with low apelin expression. An *in vitro* study found that GC cells treated with apelin showed increased migration and invasion abilities. Apelin stimulation also induced expression of APJ receptor and matrix metalloproteinase-1 (MMP-1), matrix metalloproteinase-9 (MMP-9), IL-1, and IL-6, which are associated with tumor invasion and metastasis (Feng et al., 2016). Moreover, inhibition of *APLNR* by siRNA reduced the proliferation rate, migration, and invasion abilities of GC cells, suggesting a role for the apelinergic system in the progression of gastric cancer (Feng et al., 2016).

APJ protein tissue expression might be used as a biomarker to predict therapy response and prognosis in GC patients receiving chemoradiotherapy and treatment using endostar—a modified recombinant human endostatin-since these patients with a poor response had a dramatically increased APJ expression compared to those with good treatment response (Hao et al., 2017). Apelin could also be used as a predictive biomarker for other cancer therapies. In colorectal cancer, a high concentration of apelin predicted poor response to bevacizumab therapy (Zuurbier et al., 2017). In human colon adenomas and adenocarcinomas, as well as in colon cancer cell lines, preproapelin peptide and APJ protein were overexpressed. The exogenous apelin peptides, [Pyr1]-apelin-13,−13, and −36 activate the APJ receptor, which inhibits adenylyl cyclase activity in colon cancer cells. Furthermore, apelin peptides had a protective effect against colon cancer cell apoptosis induced by pro-apoptotic agents. Interestingly, apelin peptides did not increase the proliferation of the colon cancer cells, whereas it did stimulate the phosphorylation of Akt kinase (Picault et al., 2014), whereas in human colon cancer cell line LS180, administration of apelin-13 stimulated proliferation *via* the JAG-1/Notch3 signaling pathway (Chen et al., 2017a).

Apelin has a mitogenic ability also in prostate cancer. Treatment with apelin-13 resulted in increased proliferation of prostate cancer cell lines (Tekin et al., 2014). Additionally, the 3′UTR of the *APLN* mRNA was complementary to miR-224, which might act as a tumor suppressor in human prostate cancer, suggesting that apelin is a direct target of miR-244. The knockdown of *APLN* in prostate cancer cells resulted in the abolished effect of miR-224, including inhibition of migration and invasion. Additionally, negative correlation between miR-244 and *APLN* expression levels have been reported. Downregulation of miR-244 and upregulation of *APLN* correlated with aggressiveness of tumor progression in patients with prostate cancer (Wan et al., 2015).

In patients with endometrial cancer the level of serum apelin-36 was significantly elevated and correlated with BMI (body mass index) and fasting insulin levels. However, the level of apelin-36 was not associated with tumor grade or size (Altinkaya et al., 2015).

Adipokines might activate different signaling pathways, including AMPK, PI3K/Akt, and PPARs, that might play crucial roles in the development of ovarian cancer (Dupont et al., 2012). Hoffmann et al. demonstrated, that apelin-13 could act as a mitogenic factor through the PPAR pathway in ovarian cancer cells. Furthermore, apelin-13 exhibited endocrine and autocrine actions in epithelial ovarian cancer (Hoffmann et al., 2017). In postmenopausal breast cancer patients, the serum apelin-36 level was increased compared to the control group and was positively correlated with BMI (Salman et al., 2016). In multiple myeloma patients, plasma apelin level was significantly increased, relative to a healthy control group (Maden et al., 2017).

Apelin mRNA was detected in clear cell renal cell carcinoma tissue, but there were no significant changes between cancer and normal tissue (Zhang et al., 2017). The patients with hyponatremia, a chronic kidney diseases, had increased serum apelin level, which was associated with greater risk of cancer progression and death. These data suggest, that apelin could be useful for this type of cancer prognosis (Lacquaniti et al., 2015).

In oral squamous cell carcinoma tissue the expression of apelin-36 was very weak. Moreover, apelin expression did not correlate with overall survival of patients. Stimulation of oral cancer cells with apelin-13 *in vitro* resulted in increased phosphorylation of ERK kinase. Additionally, apelin stimulated proliferation and migration of oral cancer cells (Heo et al., 2012).

A significant increase in apelin mRNA expression was also observed in glioblastoma tissue samples. Furthermore, inhibition of the apelin receptor resulted in a reduction in tumor size, vascularization, proliferation, and an increase in apoptosis (Harford-Wright et al., 2017).

In summary, apelin and its receptor are present in many types of cancer. In most cases, the levels of apelin/APJ mRNA or peptide/protein are elevated in comparison to healthy control. Additionally, the apelinergic system might contribute to cancer development. Many results suggest that the apelin/APJ system is involved in regulation of the proliferation, migration, and invasion abilities of cancer cells, leading to metastasis. Moreover, apelin plays a role in pathological angiogenesis and protects against apoptosis under tumor progression.

## Apelin in obesity-related cancer

Obesity is a condition that might increase the risk of cancer development. Storage of excess calories in the form of lipid results in extensive endocrine signaling from adipose tissue to the rest of the body. This connection is possible through adipokines secretion into the bloodstream, which connects with other metabolic organs. Therefore, it is likely that adipokines have a role in cancer development (Zhang et al., 2017). Numerous studies have demonstrated that increased BMI is associated with several types of cancer, such as prostate cancer, breast cancer, and esophageal adenocarcinoma (Paz-Filho et al., 2011). Interestingly, obesity regulates the expression of the genes connected with carcinogenesis. In the breast cancer cells of rats with diet-induced obesity, higher fold changes were detected in the expression of genes related to cellular proliferation, such as aldehyde dehydrogenase 3 family member A1 and MYC proto-oncogene. Also, the expression of the genes that protect from oncogenesis was modulated. The expression level of sirtuin-1, tensin homolog, and TGF-β were downregulated, whereas glutathione S-transferase Mu 2 and tumor protein p53 gene expression were upregulated in diet-induced obesity rats (Crujeiras et al., 2016). The level of the apelin peptide is elevated in several cancer types connected with adiposity. In obese men with colon cancer, the level of plasma apelin-12 is increased compared to a non-obese control (Al-harithy and Al-otaibi, 2015). In obese women with endometrial and breast cancer, the level of apelin-36 is also increased and positively correlated with BMI, fasting insulin levels, metabolic changes in fat tissues, hyper-inflammation, and neovascularization (Altinkaya et al., 2015; Cabia et al., 2016; Salman et al., 2016).

## Conclusions

**Figure 2:**
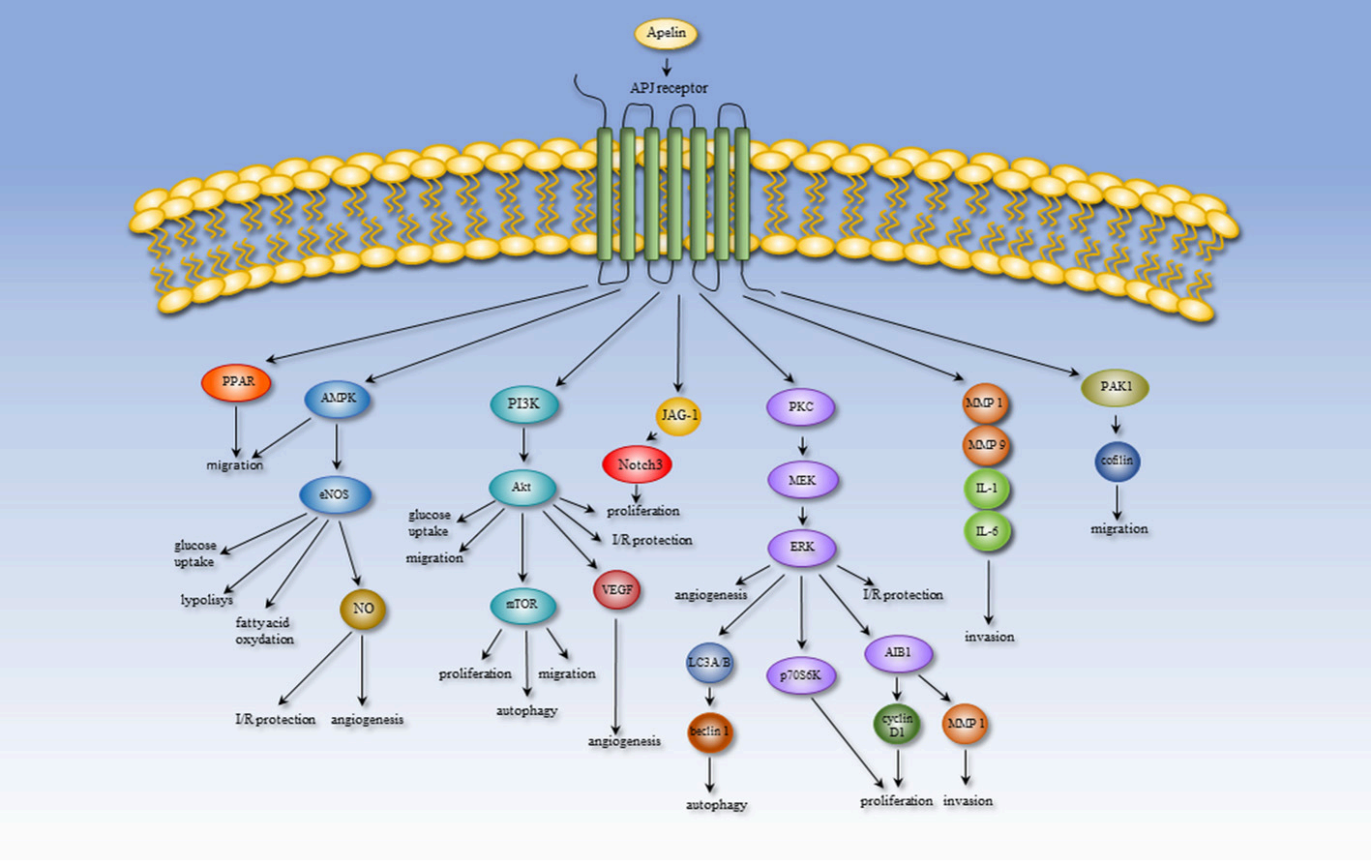
An overview of the apelin-induced signaling pathways.

Expression of apelin/APJ occurs widely in many tissue types, indicating the involvement of the apelin/APJ system in numerous physiological processes, such as angiogenesis, energy metabolism, and the regulation of fluid homeostasis and cardiovascular system. However, alternation of the microenvironmental conditions leading to the pathological process might produce a shift in the role of apelin. Therefore, the apelinergic system can participate in some pathologies, including heart failure, hypoxia-related diseases, obesity, diabetes, and cancer. The importance and effect of the apelin/APJ system are altered under pathological conditions. In failing human heart, apelin has a cardioprotective effect against myocardial infarction. Elevated apelin expression increases cardiac output, lowers blood pressure, and attenuates oxidative stress and hypertrophy. Moreover, this peptide can be treated as a biomarker for cardiovascular diseases. During hypoxia, apelin acts as a protector against apoptosis and increases the activity of antioxidant enzymes reducing oxidative stress. This peptide is also involved in hypoxia- and cancer-related angiogenesis. Secreted by adipose tissue apelin might contribute to obesity-related disorders and diabetes mellitus. Altered serum apelin levels have been detected in multiple tissues under obesity and diabetes and could be a therapeutic target in the treatment of this pathologies. Furthermore, apelin serum level is positively correlated with BMI and could increase the risk of cancer development. The role of apelin in various processes is probably mediated through several signaling pathways. Processes leading to metastasis, migration and invasion are mediated through the PPAR, PI3K/Akt/mTOR, MAPK, and PAK1/cofilin pathways. The apelinergic system also has an influence on processes connected with energy metabolism, including glucose uptake, lipolysis and fatty acid oxidation, *via* the AMPK/eNOS and PI3K/Akt pathways. The AMPK/eNOS, PI3K/Akt, and MAPK pathways could mediate angiogenesis and I/R protection. An overview of the apelin-induced signaling pathways is shown in Figure 2. Regulation of progression of tumor growth and metastasis is the most recently discovered function of apelin. Many data indicate that in multiple cancer types, apelin and its receptor might be used as a prognostic biomarker. However, in many studies, the available results are unclear. First, the expression changes of apelin/APJ mRNA do not correlate with its serum concentrations. Moreover, not all forms of apelin peptides are examined, or there is no distinction between peptide types. Additionally, the antibodies used in experiments are often non-specific and recognize more than one form of apelin. Interestingly, apelin receptor antagonists could be promising therapeutic compounds for cancer treatment. Nevertheless, the most often used antagonist—ML221—could also inhibit more receptor types (e.g., kappa opioid or the benzodiazepinone receptors) (Maloney et al., 2012). This result suggests that ML221 could inhibit another receptor and act through different signaling pathways. After appraising the available data, we propose that there remains much to learn about the role of apelin in pathological processes.

## Author contributions

MW, KP-G, and DN contributed conception and design of work. MW wrote the first draft of the manuscript. All authors contributed to manuscript revision, read and approved the submitted version.

### Conflict of interest statement

The authors declare that the research was conducted in the absence of any commercial or financial relationships that could be construed as a potential conflict of interest.
